# Gingival Enlargement Can Constitute the Only Diagnostic Sign of Leukemia: Report of an Unusual Case

**DOI:** 10.7759/cureus.47959

**Published:** 2023-10-30

**Authors:** Vasileios Zisis, Stefanos Zisis, Eleuftherios Anagnostou, Nikolaos Dabarakis, Athanasios Poulopoulos, Dimitrios Andreadis

**Affiliations:** 1 Oral Medicine/Pathology, Aristotle University of Thessaloniki, Thessaloniki, GRC; 2 Periodontology, Rheinisch-Westfälische Technische Hochschule Aachen (RWTH) Aachen University, Aachen, DEU; 3 Dentoalveolar Surgery, Implantology and Oral Radiology, Aristotle University of Thessaloniki, Thessaloniki, GRC

**Keywords:** acute myeloid leukemia ( aml), cd117, cd68, cd45, gingival overgrowth, leukemia

## Abstract

Gingival hyperplasia may arise due to microbial-related local irritation, mouth breathing, drug administration, genetic disorders, leukemia, Wegener granulomatosis, Crohn’s disease, and sarcoidosis. The background may be inflammatory, fibrotic, or combined. The aim of this study was to present the diagnostic procedure for a case of gingival enlargement, which was the only sign of a severe systemic disease in a young male adult. The patient was referred, complaining of persistent gingival bleeding in the posterior area of the maxilla, bilaterally. Clinically, a diffuse gingival enlargement was noticed without regional lymphadenopathy. The histopathological examination revealed abundant neoplastic cells of hemopoietic origin with strong and diffuse positivity for CD45 and CD68, and in addition, scattered neoplastic cells exhibited mild to moderate positivity for c-kit (CD117), indicating the diagnosis of acute myeloid leukemia, which diffusely infiltrated the lamina propria of the gingiva. The numerous conditions leading to gingival enlargement other than gingivitis or periodontitis are a diagnostic challenge in clinical practices. From this point of view, the role of the dentist is crucial when commencing the diagnosis of severe systemic diseases like leukemia.

## Introduction

Gingival hyperplasia may arise due to local irritation, mouth breathing, drug administration, genetic disorders, leukemia, Wegener granulomatosis, Crohn’s disease, and sarcoidosis [1]. Drug-induced gingival hyperplasia is primarily associated with anticonvulsants such as phenytoin, immunosuppressants such as cyclosporine A, calcium channel blockers such as nifedipine, verapamil, diltiazem, contraceptives and monoclonal antibodies [2]. The background may be inflammatory, fibrotic, or combined [1]. Leukemia is classified as a malignant neoplasm that impacts the hematolymphoid system, leading to the uncontrolled proliferation of neoplastic cells [3,4]. The global incidence of leukemia in 2017 was estimated to be approximately 2.43 million cases, with an age-standardized prevalence rate of 32.26 per 100,000 individuals [5]. Leukemias rank as the 15th most prevalent form of malignancy [6]. Leukemia manifests in two separate forms: either as precursor cells, which are immature, or as mature cells. These forms give rise to acute or chronic leukemia, each with a unique prognosis based on the age of the individual affected. The illness is frequently categorized into four primary subgroups, namely acute lymphoblastic leukemia (ALL), acute myeloid leukemia (AML), chronic lymphoblastic leukemia (CLL), and chronic myeloid leukemia (CML) [4,7]. Individuals affected by blood diseases commonly present with systemic manifestations, including weight loss, fever, and fatigue due to anemia, neutropenia, and thrombocytopenia [4,7-9]. Blood diseases have the potential to impact several anatomical sites, encompassing the oral and oropharyngeal areas [3,10]. Oral symptoms are primarily reported in patients afflicted with acute types of leukemia [11]. Specifically, over 30% of individuals who are diagnosed with leukemia experience various oral manifestations [12]. The most commonly observed findings include gingival hypertrophy, spontaneous bleeding, petechiae, ulcerations, and oral mucosa pallor [11,13]. The initial indications of leukemia often become apparent in the oral cavity as a result of the invasion of leukemic cells in oral tissues or the reduction of normal bone marrow elements (red cells, white blood cells, and platelets), particularly during the acute stage of the illness [14]. The aim of this study is to present a case of gingival enlargement due to acute lymphoblastic leukemia in a young male adult as the only clinical sign that led to diagnosis.

## Case presentation

**Figure 1:**
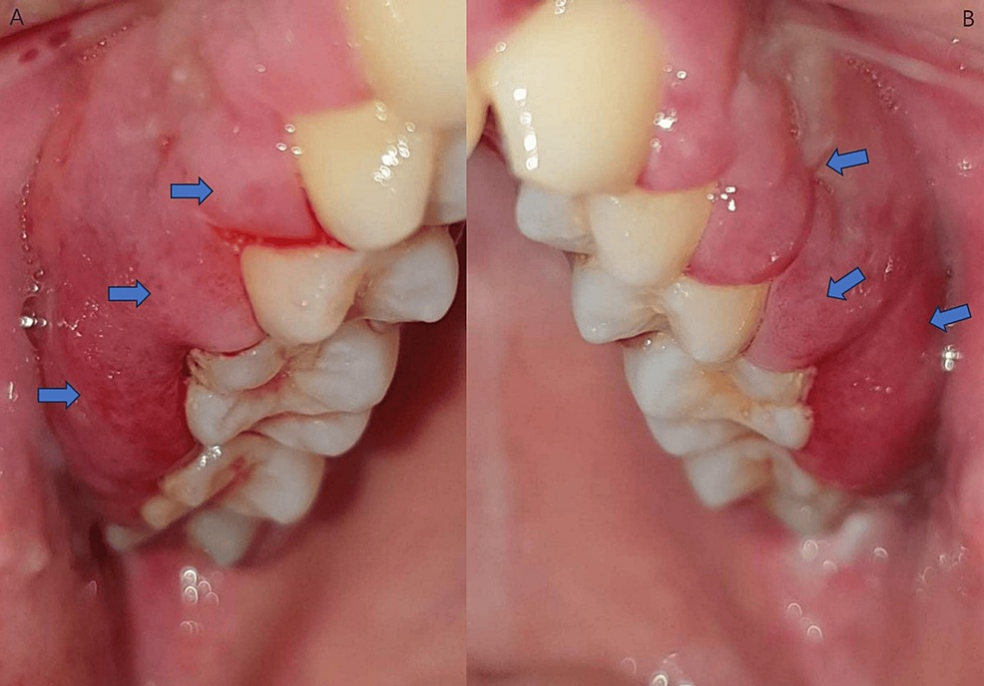
Gingival enlargement is indicated by the blue arrows in the first quadrant (A) and the second quadrant (B).

A male patient, 26 years old, was referred by his dentist to the Department of Oral Medicine and Pathology, School of Dentistry, Aristotle University of Thessaloniki, Greece, complaining of persistent gingival bleeding (which commenced one week before the patient was eventually referred) in the posterior area of the maxilla, bilaterally, and mild pain due to food impaction in the interdental area. Before the examination, the patient provided written informed consent. This form was approved by the School of Dentistry, Aristotle University of Thessaloniki, and was in accordance with the Helsinki Declaration for research and patient ethics. Subsequently, the patient was examined thoroughly. The physical examination revealed diffuse reddish swelling, palpable gingival masses, bleeding on probing, and the presence of pseudopockets of 4-6mm (Figure 1).

**Figure 2:**
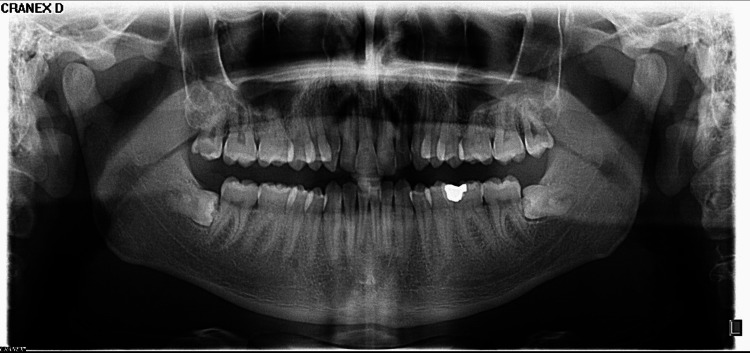
OPG examination.

The patient was then referred for both OPG and blood examinations. The OPG didn’t include any pathological findings (Figure 2).

**Figure 3:**
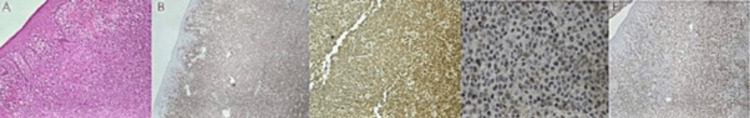
Histopathological examination. A. Acute myeloid leukemia neoplastic cells diffusely infiltrate the lamina propria of the gingiva (H-E staining X100). B. Strong and diffuse positivity of neoplastic cells for CD45 (LCA) (immunoperoxidase staining X40). C. Tumor cells are diffusely and strongly positive for CD68 (immunoperoxidase staining X100). D. Scattered neoplastic cells exhibit mild to moderate positivity for c-kit (CD117) (immunoperoxidase staining X400). E. Acute myeloid leukemia is characterized by extremely high nuclear proliferation, with Ki-67 index positive in 90% of lesional cells (immunoperoxidase staining X40).

Interestingly, the blood tests revealed a WBC count of 42.6 K/μl (normal range 4-10 K/μl), including monocytes 24.9 K/μl (normal range 2-12 K/μl), neutrophils 19 K/μl (normal range 1.4-7 K/μl), red blood cells 2.9 M/μl (normal range 4.7-6.1 M/μl), hemoglobin 9.1 g/dL (normal range 14-18 g/dL), and hematocrit 25.9% (42-52%). Based on these findings, a biopsy was carried out. The histopathological examination revealed abundant neoplastic cells of hemopoietic origin that diffusely infiltrate the lamina propria of the gingiva, bearing strong and diffuse positivity for CD45 and CD68. In addition, scattered neoplastic cells exhibited mild to moderate positivity for c-kit (CD117). All these findings led to a diagnosis of acute myeloid leukemia, and the Ki-67 positivity in 90% of malignant cells was an indicator of an extremely high cellular proliferation capacity, as expected (Figure 3).

## Discussion

Acute myeloid leukemia (AML) is a malignancy characterized by its extreme aggressiveness [15]. It accounts for roughly 25% of cases of leukemia in the pediatric population [16]. Therefore, in our case, the age of the patient may be considered atypical. The significant mortality rate underscores the crucial importance of a precise and expeditious diagnosis [15]. The findings have the potential to forecast the response to treatment and evaluate the level of risk [17]. The health state of the oral cavity is closely indicative of the overall health of the patient. The detection and recognition of the signs and symptoms of oral lesions can serve as an indicator of underlying and significant systemic involvement. Oral lesions have the potential to serve as the initial manifestation of acute leukemia, therefore making them a significant diagnostic signal [18]. Identifying these lesions can potentially facilitate the diagnosis of acute myeloid leukemia (AML). The prevalence of oral symptoms is significantly higher in cases of myeloid and monocytic/monoblastic leukemia [18,19]. Petechiae, or spontaneous gingival bleeding, are observed in 56% of patients, whereas ulcerations are present in 53%, and gingival enlargement is reported in 36% of the patients [15,18]. The aforementioned characteristics are the prevailing primary diagnostic indications of leukemia. In particular, gingival infiltration, as in our case, constitutes the initial manifestation of acute myeloid leukemia (AML) in 5% of the patients, which is more commonly observed in the myelomonocytic and monocytic subtypes of leukemia [15]. It is important to acknowledge however infrequently, the gingiva can serve as a site of extra-medullary localization, referred to as myeloid sarcoma, which may occur during relapse as well [15]. This phenomenon is observed in approximately 3%-5% of patients with acute myeloid leukemia (AML), with a higher incidence of involvement of the skin, bones, or gastrointestinal tract.

The initial manifestations of pancytopenia, which encompass anemia, neutropenia, or thrombocytopenia, may include non-specific indications such as fatigue, dyspnea, fever, pallor, weight loss, or bleeding [14]. In this case, the patient didn’t report any of these symptoms apart from the gingival enlargement and bleeding. Consequently, various organs or lymph nodes may be infiltrated by neoplastic cells, leading to the development of hepatosplenomegaly, lymphadenopathy, bone discomfort, etc. The involvement of the testicular and central nervous system (CNS) [20], as well as the involvement of the lymphoid-bearing tissue located in the orofacial area, which includes the tonsils, may be noticed [14]. In the oral cavity, one may observe mucosal pallor, gingival hemorrhage, or ecchymoses, non-specific ulcerations, and opportunistic infections, whereas the presence of lymphadenopathy in the head and neck area is a persistent indication [14]. In our case, palpation didn’t reveal any regional lymphadenopathy, indicating that the diagnosis took place at an early stage. Regional lymphadenopathy and an elevated WBC count constitute negative prognostic factors as well as indicators of chronicity [21]. Pericoronitis may also be observed [22]. A case is also documented where trismus was observed as the initial manifestation of acute leukemia in a six-year-old male patient [23]. Despite conducting an intraoral examination and panoramic radiography, no indications of infection or other pathological conditions were found. Trismus can be described as a significant infiltration of leukemic cells into the deep muscles involved in mastication [23]. The mucosal abnormalities may encompass the presence of extensive ulcers, a coated tongue, fetor oris, shallow papillae, tender oral mucosa, and oral mucosal infections such as mucositis, candidiasis, herpes simplex, varicella/zoster, and cytomegalovirus [14]. The background of gingival enlargement and its wide variety of possible diagnoses render the establishment of a common local therapeutic approach difficult. The presence of microbial plaque induces inflammation and enhances gingival enlargement, regardless of its primary causative factor, thus necessitating the application of oral hygiene measures [14]. In cases of drug-induced gingival hyperplasia, anticonvulsants such as phenytoin, immunosuppressants such as cyclosporine A, calcium channel blockers such as nifedipine, verapamil, diltiazem, contraceptives and monoclonal antibodies may have to be replaced or substituted [2].

## Conclusions

Gingival hyperplasia constitutes the crossroads of a broad spectrum of oral and systematic diseases, and its accurate diagnosis may prove to be tricky. Typically, initial indications of leukemia become apparent within the oral cavity, leading patients to frequently seek dental treatment under the assumption that the ailments are of a localized nature. The dentist plays a crucial role in the timely identification of hematologic disorders. It is noteworthy to mention that dentists have a significant role in commencing the diagnosis of leukemia in cases of oral manifestations. Therefore, it is imperative that these manifestations are easily identifiable, prompting a comprehensive inquiry involving supplementary tests or a referral to a qualified practitioner to arrive at a definitive diagnosis.
